# Supplementary material for: Religious leaders’ perspectives on childhood immunization in Bauchi State, Nigeria: A Qualitative Study
Source: medRxiv. 2026 Apr 4:2026.02.19.26346395. Preprint. [Version 2] doi: 10.64898/2026.02.19.26346395 (PMC13060459; doi:10.64898/2026.02.19.26346395)
Supplement: 1 [file NIHPP2026.02.19.26346395V2-supplement-1.pdf]

medRxiv preprint doi: https://doi.org/10.64898/2026.02.19.26346395; this version posted April 4, 2026. The copyright holder for this preprint (which was not certified by peer review) is the author/funder, who has granted medRxiv a license to display the preprint in perpetuity.
It is made available under a CC-BY 4.0 International license .

Supplementary File 2

Table 2. Thematic Analysis overview of Themes, Subthemes, Key Insights, and Representative Quotes (N = 22)

Themes	Subthemes	Key Insights	Illustrative quotation	Frequency (n/22)
**Knowledge**	Vaccine mechanism & benefits	Leaders commonly describe vaccines as priming immunity and preventing disease.	"Immunization weakens the virus so the body can develop resistance…" (Participant 4)	most (19/22)
	Economic perspective	Immunization is linked to fewer clinic visits and household cost savings.	"We no longer visit the hospital like before… the money… is now used for the education of our children." (Participant 2)	some (8/22)
	Community perceptions	Leaders reported increasing community acceptance and eagerness to vaccinate children.	"Once [immunization] is announced… you will see that people are eager to take their children." (Participant 11)	some (6/22)
	Personal experience	Past illness experiences reinforce perceived vaccine benefits.	"My first daughter suffered measles… I have seen the positive impact." (Participant 6)	most (18/22)
**Beliefs**	Religious alignment	Immunization was framed as compatible with Islam and, in the single Christian interview, also as compatible with Christianity.	"Knowledge is given by God… anything that benefits humanity, we practice it" (Participant 4)	most (18/22)

medRxiv preprint doi: https://doi.org/10.64898/2026.02.19.26346395; this version posted April 4, 2026. The copyright holder for this preprint (which was not certified by peer review) is the author/funder, who has granted medRxiv a license to display the preprint in perpetuity.
It is made available under a CC-BY 4.0 International license .

	Misconceptions & misinformation	Population control rumors and suspicion of free house-to-house (H2H) delivery persist.	"People say Westerners intend to prevent childbirth… [so] Muslims should never trust them." (Participant 3)	some (6/22)
	Evolving beliefs	Doubts often shift after religious and scientific explanation and observation.	"In the past I was not supporting immunization… now I realize it is beneficial." (Participant 7)	many (11/22)
	Skepticism	Few express non-doctrinal distrust or indecision.	"We can't say it is haram… but I just don't have faith in it." (Participant 20)	few (3/22)
**Attitudes**	Familial influence & early exposure	Parental modelling and clerical lineage shape durable positive views.	"Even when I was small my father immunized me… I don't think I'll change." (Participant 4)	some (10/22)
	Advocacy & support	Leaders provide visible endorsements to signal safety and effectiveness.	"We set up a vaccination center… I was the first to be publicly vaccinated." (Participant 1)	few (5/22)
	Community acceptance vs resistance	Acceptance is rising, but a resistant subset requires mediation.	"Some accept, others reject… even after leaders intervene." (Participant 3)	few (3/22)

medRxiv preprint doi: https://doi.org/10.64898/2026.02.19.26346395; this version posted April 4, 2026. The copyright holder for this preprint (which was not certified by peer review) is the author/funder, who has granted medRxiv a license to display the preprint in perpetuity.
It is made available under a CC-BY 4.0 International license .

	Doubts overcome via religious explanation	Scriptural clarification shifts peers' attitudes.	"We explained what the Prophet said… from there some changed." (Participant 2)	some (7/22)
	Perceived neglect vs prevention	Frustration with curative care gaps fuels resentment toward vaccines.	"When they are sick and go to the hospital, no one cares for them, but now they are being disturbed with vaccines." (Participant 10)	few (3/22)
	Barriers to uptake	Side effects, queues, distance, cost, and distrust of foreign motives noted.	"They are suffering from hunger… [yet] it is always about vaccination." (Participant 18)	most (20/22)
	Observed community transformation	Leaders report fewer common illnesses and perceived growing acceptance.	"Diseases that were once common have reduced drastically." (Participant 1)	few (4/22)
**Practices**	Delivery preference H2H	Strong preference for H2H due to time or transport barriers, with clinic-based RI also widely used.	"If we are met at home, we allow it… we cannot go to the hospital to suffer" (Participant 9)	most (18/22)

medRxiv preprint doi: https://doi.org/10.64898/2026.02.19.26346395; this version posted April 4, 2026. The copyright holder for this preprint (which was not certified by peer review) is the author/funder, who has granted medRxiv a license to display the preprint in perpetuity.
It is made available under a CC-BY 4.0 International license .

	Personal and family immunization	Leaders reported ensuring their children's vaccinations and, where eligible (e.g., COVID-19), their own.	"Even when we were young, we received immunization… I myself do take my wife to hospital for… immunization" (Participant 6)	most (18/22)
	Religious-leader roles	Activities include mobilization, sensitization, escorting, and hosting immunization activities.	"We visited remote areas to sensitize people about benefits." (Participant 2)	most (20/22)
	Community mobilization	Messaging via mosques, schools, associations is common.	"I took my campaign to mosques, Islamic schools, community groups…" (Participant 13)	many (14/22)
	Non-uptake and refusal	A small minority reported not accepting immunization on religious grounds.	"I won't accept anything that is not sharia… what God has planned will always find its way." (Participant 20)	few (1/22)
**Recommendations**	Communities and parents	Seek accurate information and verify doubts with authorities.	"If you see a negative result, contact the government for clarification." (Participant 6)	few (5/22)

medRxiv preprint doi: https://doi.org/10.64898/2026.02.19.26346395; this version posted April 4, 2026. The copyright holder for this preprint (which was not certified by peer review) is the author/funder, who has granted medRxiv a license to display the preprint in perpetuity.
It is made available under a CC-BY 4.0 International license .

	Health workers	Be courteous, partner with rural clerics, arrive on time, follow up.	"…use kind words and be courteous." (Participant 17)	some (6/22)
	Religious leaders	Lead by example and disseminate verified permissibility or benefits to peers and congregants.	"Practice what you preach…people look to your opinion." (Participant 1)	few (3/22)

Notes: Participant frequency (n/22) indicates the number of unique participants who contributed at least one coded excerpt to the subtheme. This count is provided to convey the breadth of views within this sample. Qualitative frequency labels were defined as: few = 1–5/22 (<25%); some = 6–10/22 (25–<50%); many = 11–16/22 (50–<75%); most = 17–22/22 (≥75%). Quotations are lightly edited for readability; ellipses indicate omitted text; bracketed text indicates clarifications. Abbreviations**:** H2H, house to house; RI, routine immunization; Participant #, participant ID.
